# Supplementary material for: Correcting Artifacts in Ratiometric Biosensor Imaging; an Improved Approach for Dividing Noisy Signals
Source: Front Cell Dev Biol. 2021 Aug 20;9:685825. doi: 10.3389/fcell.2021.685825 (PMC8418531; doi:10.3389/fcell.2021.685825)
Supplement: Supplementary file 1 [file Data_Sheet_1.pdf]

Supplementary Information for

Correcting artifacts in ratiometric biosensor imaging; an improved approach for dividing noisy signals

Daniel J. Marston[2], Scott Slattery[2], Klaus M. Hahn[2,4,*], Denis Tsygankov[1,*]

[1]Wallace H. Coulter Department of Biomedical Engineering, Georgia Institute of Technology and Emory University, Atlanta, GA.
[2]Department of Pharmacology, University of North Carolina, Chapel Hill, NC.
[4]Lineberger Comprehensive Cancer Center, University of North Carolina, Chapel Hill, NC.

* To whom correspondence should be addressed
Email: denis.tsygankov@bme.gatech.edu

This PDF file includes:

Supplemental Text

Extended derivation of the mathematical expression in section "*A hypothetical cell model illustrating effects of noise on ratio values*":

$$\frac{s_F x + b_F + n_F}{s_D x + b_D + n_D} = \frac{s_F\left(x + \frac{b_D}{s_D}\right) + \left(b_F - s_F\frac{b_D}{s_D}\right) + n_F}{s_D\left(x + \frac{b_D}{s_D}\right) + n_D}$$

Here, the first step is to add $\left(s_F\frac{b_D}{s_D} - s_F\frac{b_D}{s_D}\right)$, which is **zero**, to the numerator and multiply b_D by $\frac{s_D}{s_D}$, which is **one**, in the denominator. Second step is combining terms. The last step is taking s_F and s_D out of brackets:

$$\frac{s_F x + b_F + n_F}{s_D x + b_D + n_D} = \frac{s_F x + \left(s_F\frac{b_D}{s_D} - s_F\frac{b_D}{s_D}\right) + b_F + n_F}{s_D x + \frac{s_D}{s_D}b_D + n_D} = \frac{s_F x + s_F\frac{b_D}{s_D} - s_F\frac{b_D}{s_D} + b_F + n_F}{s_D x + \frac{s_D}{s_D}b_D + n_D}$$

$$= \frac{\left(s_F x + s_F\frac{b_D}{s_D}\right) + \left(-s_F\frac{b_D}{s_D} + b_F\right) + n_F}{\left(s_D x + \frac{s_D}{s_D}b_D\right) + n_D} = \frac{s_F\left(x + \frac{b_D}{s_D}\right) + \left(b_F - s_F\frac{b_D}{s_D}\right) + n_F}{s_D\left(x + \frac{b_D}{s_D}\right) + n_D}$$

Extended derivation of the mathematical expression in section "*Use of a noise correction factor; identification and correction of artifacts without using direct background subtraction*":

$$Ratio(x,y) = \frac{image1(x,y)}{image2(x,y)} = \frac{a_0(S_2(x,y) + B_2) + (B_1 - a_0B_2) + N_1(x,y)}{(S_2(x,y) + B_2) + N_2(x,y)}$$

Here, the first step is to add $(a_0B_2 - a_0B_2)$, which is **zero**, to the numerator and combine the terms as highlighted in cyan and gray. The last step is taking a_0 out of brackets:

$$Ratio(x,y) = \frac{image1(x,y)}{image2(x,y)} = \frac{a_0S_2(x,y) + B_1 + N_1(x,y)}{S_2(x,y) + B_2 + N_2(x,y)}$$

$$= \frac{a_0S_2(x,y) + a_0B_2 - a_0B_2 + B_1 + N_1(x,y)}{S_2(x,y) + B_2 + N_2(x,y)}$$

$$= \frac{a_0S_2(x,y) + a_0B_2 - a_0B_2 + B_1 + N_1(x,y)}{S_2(x,y) + B_2 + N_2(x,y)}$$

$$= \frac{a_0(S_2(x,y) + B_2) + (B_1 - a_0B_2) + N_1(x,y)}{(S_2(x,y) + B_2) + N_2(x,y)}$$
